# Mystery of the lost gallstone‐Part 1

**DOI:** 10.1002/ccr3.3129

**Published:** 2020-07-16

**Authors:** Aikaterini Paraskeva, Alexandros Triantafyllidis, Konstantinos A. Boulas, Maria Nathanailidou, Konstantinos Chatzipourganis, Anestis Hatzigeorgiadis

**Affiliations:** ^1^ Department of General Surgery General Hospital of Drama Drama Greece

**Keywords:** abscess, cholecystectomy, complication, gallstones, Laparoscopy, perforation

## Abstract

If gallbladder perforation occurs during laparoscopic cholecystectomy, every spilled gallstone should be retrieved to minimize possible late gallstone‐related septic complications.

## Graphical abstract

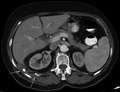

## 1 INTRODUCTION

**Figure 1:**
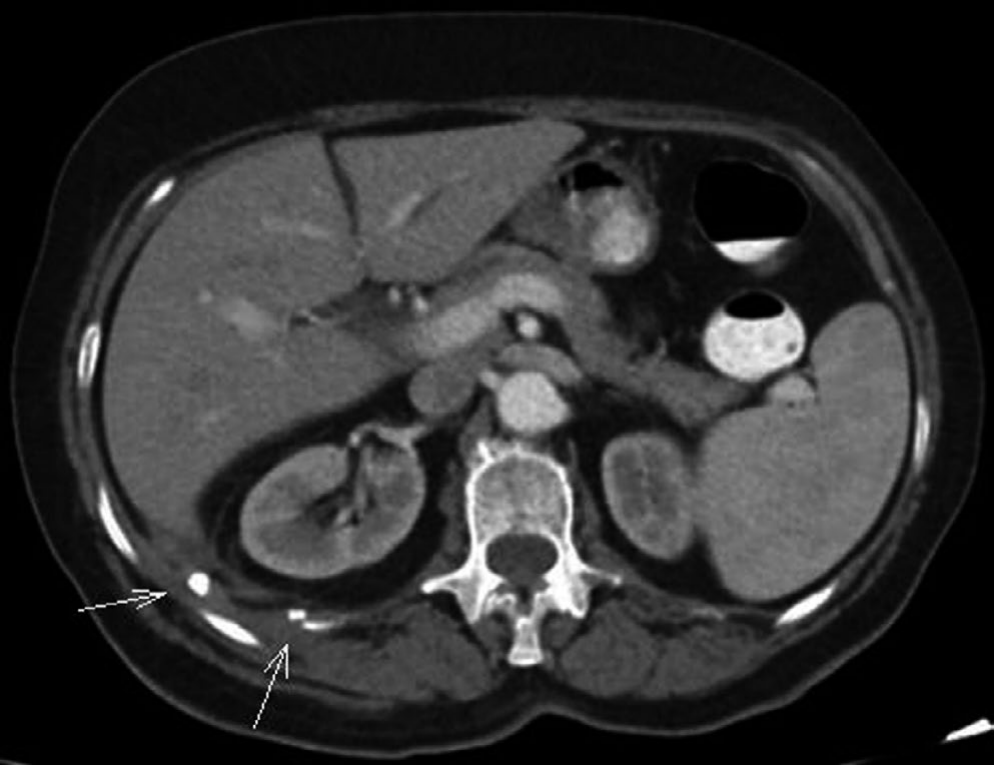
CT revealed the presence of two small 10 and 7 mm hyperattenuating lesions (white arrows) at the edge of the Morrison's pouch behind the upper pole of the right kidney with no evidence of intraabdominal and retroperitoneal abscess formation

**Figure 2:**
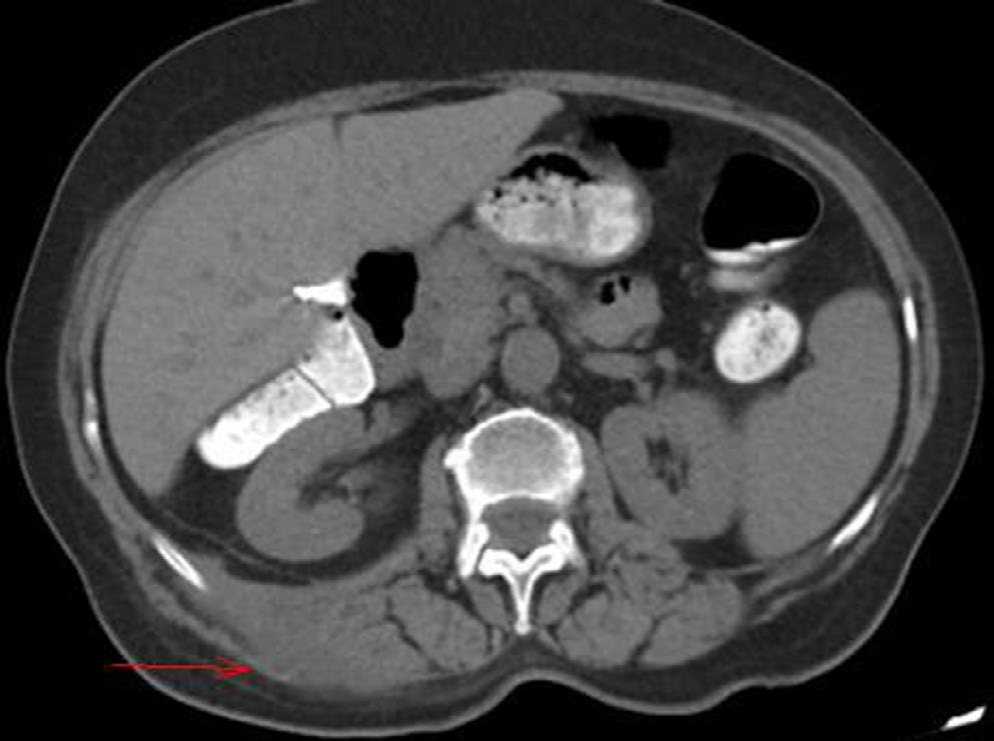
CT revealed the presence of abscess formation (red arrow) at the superficial and intermediate muscle layer of the posterior abdominal wall (latissimus dorsi, external oblique and internal oblique muscle) without involvement of the deep muscle layer (quadratus lumborum and psoas muscle)

A 66‐year‐old female patient, with a history of type II diabetes mellitus and laparoscopic cholecystectomy for chronic cholecystitis 1 year ago, presented to the emergency department complaining of nonspecific low back pain and right lumbar tumefaction over the preceding 2 months and 7 days, respectively. Clinical examination revealed the presence of a tender 6x4cm palpable soft‐tissue mass suggestive of right lumbar abscess. CT performed to delineate disease extension and etiology, as shown in Figures 1 and 2.

## 2 QUIZ QUESTION: WHAT IS YOUR DIAGNOSIS?

Taken into account the history of cholecystectomy, the two lesions in Morrison's pouch considered to be unretrieved gallstones. The right lumbar abscess considered to be late septic complication of spilled gallstones. The most common complications related to unretrieved gallstones are septic with an incidence of 0.08%‐0.3%. Time interval between cholecystectomy and septic complications ranges between 1 month and 20 years.^1^ Combination of pneumoperitoneum and irrigation can disseminate gallstones within the peritoneal cavity causing local and unusual distant complications as abdominal wall abscess, intraabdominal and retroperitoneal abscess, hepatic, splenic and greater omentum abscess, pulmonary abscess, and thoracic empyema.^2^ The patient treated with open drainage of the lumbar abscess without gallstone removal along with watch and wait approach for surgical stone removal in case of abscess recurrence.

## CONFLICT OF INTEREST

None declared.

## AUTHOR CONTRIBUTIONS

All authors equally accessed the data and contributed to the preparation of the manuscript. PA and HA were equally responsible for making and performing treatment decisions. HA reviewed the manuscript for critical intellectual content and had the final approval.

## STATEMENT OF HUMAN AND ANIMAL RIGHTS

The present article does not contain any studies with human or animal subjects performed by any of the authors.

## INFORMED CONSENT

Informed consent was obtained from the patient.
